# Combination of Circulating Tumor Cells with Serum Carcinoembryonic Antigen Enhances Clinical Prediction of Non-Small Cell Lung Cancer

**DOI:** 10.1371/journal.pone.0126276

**Published:** 2015-05-21

**Authors:** Xi Chen, Xu Wang, Hua He, Ziling Liu, Ji-Fan Hu, Wei Li

**Affiliations:** 1 Cancer and Stem Cell Center, First Affiliated Hospital, Jilin University, Changchun, Jilin 130061, P.R. China; 2 Stanford University Medical School, Palo Alto Veterans Institute for Research, Palo Alto, CA, 94304, United States of America; The Ohio State University, UNITED STATES

## Abstract

Circulating tumor cells (CTCs) have emerged as a potential biomarker in the diagnosis, prognosis, treatment, and surveillance of lung cancer. However, CTC detection is not only costly, but its sensitivity is also low, thus limiting its usage and the collection of robust data regarding the significance of CTCs in lung cancer. We aimed to seek clinical variables that enhance the prediction of CTCs in patients with non-small cell lung cancer (NSCLC). Clinical samples and pathological data were collected from 169 NSCLC patients. CTCs were detected by CellSearch and tumor markers were detected using the Luminex xMAP assay. Univariate analyses revealed that histology, tumor stage, tumor size, invasiveness, tumor grade and carcinoembryonic antigen (CEA) were associated with the presence of CTCs. However, the level of CTCs was not associated with the degree of nodal involvement (N) or tumor prognostic markers Ki-67, CA125, CA199, Cyfra21-1, and SCCA. Using logistic regression analysis, we found that the combination of CTCs with tumor marker CEA has a better disease prediction. Advanced stage NSCLC patients with elevated CEA had higher numbers of CTCs. These data suggest a useful prediction model by combining CTCs with serum CEA in NSCLC patients.

## Introduction

Lung cancer is the leading cause of cancer-related deaths worldwide [1]. Non-small-cell lung cancer (NSCLC) accounts for 75–80% of lung cancer cases. NSCLC is typically not diagnosed until the disease has reached the advanced stage, leading to low survival rates, with a 5-year survival rate of 20% [1,2]. A number of biomarkers have been used in the clinic as prognostic markers for NSCLC patients. These include carcinoembryonic antigen (CEA), cancer antigen (CA)-199, CA-125, squamous cell carcinoma antigen (SCC-Ag), and cytokeratin-19 fragments antigen 21–1 (CYFRA21-1). Elevated levels of these markers have been associated with poor prognosis [3–12]. Among them, CEA and CYFRA 21–1 are the most sensitive tumor markers in NSCLC [13,14].

Circulating tumor cells (CTCs) are tumor cells that leave the primary tumor site and enter the bloodstream, where they can spread to other organs. CTCs can be identified in the peripheral blood by histologic staining for epithelial and cancer-specific markers. Currently, the only detection kit approved by the U.S. Food and Drug Administration (FDA) is the CellSearch system (Veridex, NJ), which uses specific antibodies to identify and quantify CTCs in a 7.5 mL blood sample.

To date, the role of CTCs in NSCLC remains unclear. For example, while some studies correlate CTCs with poor prognosis [15–18], others found no correlation [19]. A recent meta-analysis of 20 studies with a total of 1576 patients revealed that the presence of CTCs was associated with poor prognosis in NSCLC patients [20]. Of note, the prognostic value of CTCs remains controversial. Previous studies have reported that about 30% of NSCLC patients have at least one CTC per 7.5 mL of blood, and about 15% of NSCLC patients have five or more CTCs per 7.5 mL of blood, with higher CTC levels in patients with distant metastases [15, 21–22]. In addition to its low sensitivity, the high cost of CTC detection has made it difficult to become a routine clinical test for NSCLC, particularly in Chinese populations.

To achieve a better understanding of the significance of CTCs in NSCLC, we have initiated a prospective, single institution study to characterize the CTCs in NSCLC patients and examined the relationship between CTCs and other clinical factors. We aimed to test the hypothesis that the presence of CTCs in combination with tumor biomarkers could better predict tumor invasiveness in NSCLC patients.

## Materials and Methods

### Study population

This study was carried out at the First Hospital of Jilin University (Changchun, Jilin, China). The study was approved by the Ethics Committee of the First Hospital of Jilin Medical University, and conducted according to Declaration of Helsinki principles. Written informed consent was obtained from all enrolled patients prior to any interventions. Patients with histologically confirmed NSCLC were eligible. Prior to treatment, Tumor Node Metastasis (TNM) staging (7th edition) was assessed by Computated Tomography (CT) scans [23].

### CTC analysis

The CellSearch system (Veridex, NJ, USA) was used to quantify CTCs in 7.5 mL blood samples drawn from patients within seven days prior to treatment (defined as baseline). Blood samples were collected in 10 mL CellSave (Veridex) preservative tubes, stored at room temperature, and processed within 96 hours of collection, according to the manufacturer’s instructions. CTCs are defined as cells with round to oval morphology, a, 4’,6-diamidino-2-phenylindole (DAPI) positive nucleus of 4 μM or greater, positive cytoplasmic staining for cytokeratins (CK-8, CK-18, and CK-19), and the absence of CD45 expression.

### Tumor marker analysis

Sera were separated from a 2 mL sample of coagulated blood from each patient. A Luminex xMAP assay (Luminex, Austin, TX, USA) was use to detect the tumor markers carcinoembryonic antigen (CEA), cancer antigen (CA)-199, CA-125, squamous cell carcinoma antigen (SCC-Ag), and cytokeratin-19 fragments antigen 21–1 (CYFRA21-1). The level of each marker was compared to the normal reference values of 5 ng/mL CEA, 35 U/mL CA19-9, 35 U/mL CA125, 1.5 ng/mL SCC, and 5 ng/mL CYFRA21-1. Due to the limit of serum volume, only 109 patients had their serum analyzed for CEA levels, 70 patients for CA125, 76 patients for CA199, 107 patients for CYFRA21-1, and 75 patents for SCCA.

### Pathology specimen and immunohistochemistry

Tumor specimens were obtained from patients with stage IIIB or IV NSCLC for pathological analysis. Tumor samples were fixed in formalin, embedded in paraffin, cut into 4 mm sections, and affixed to glass slides. Cell proliferation was assessed by immunohistochemistry using a monoclonal antibody against Ki-67 (MAIXIN-BIO Inc., China). Ki-67 staining in fewer than 25% of cells was considered negative, whereas staining in > 25% of cells was considered to be Ki-67 positive [24].

### Statistical analysis

Individual variables were assessed by univariate analysis using the Chi squared test. Risk ratios were calculated for each variable to assess the predictive value for CTCs. Logistic regression analysis was used to assess the relationships between CTC counts and clinico-pathological data. All analyses were conducted by using SPSS v19.0 software (SPSS, Inc., Chicago, IL, USA). For all analyses, a *p*-value less than 0.05 was considered statistically significant.

## Results

### Patient characteristics

**Fig 1:**
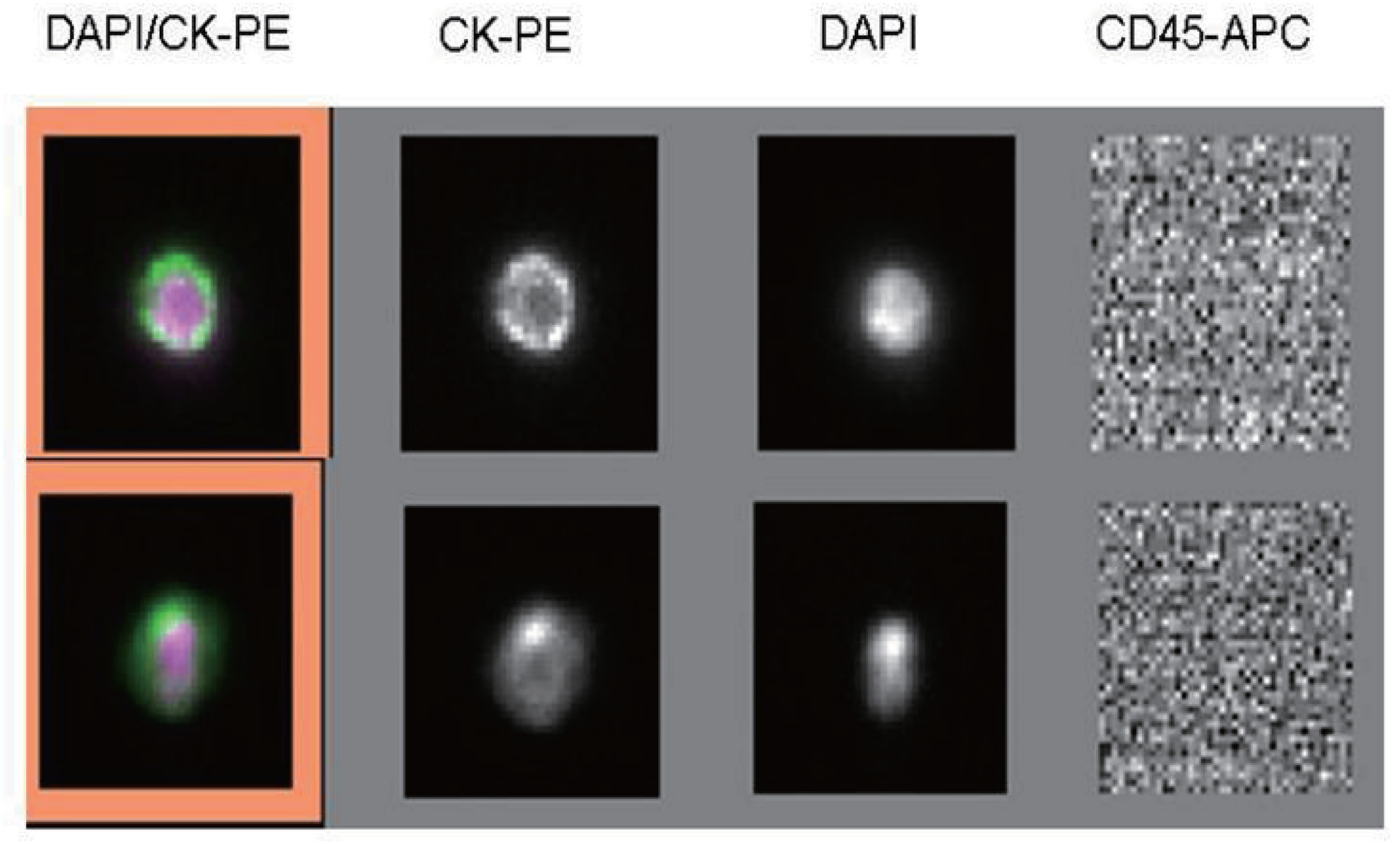
Immunostaining of a single lung cancer CTC isolated from patient peripheral blood. Positive immunomagnetic selection with anti-EpCAM Ab was followed by morphological confirmation with staining for cytokeratins (cytoplasm), DAPI (nucleus), and CD45 (negative).

One hundred and sixty-nine patients with NSCLC were recruited between July 2012 and January 2014, and their characteristics were recorded (Table 1). CTCs were quantified from a 7.5 mL blood sample for each patient (Fig 1). The median follow-up duration was 1.1 years. Thus, survival analysis would not be conducted on this cohort population. Overall, the prediction of NSCLC by CTCs was relatively low in our cohort samples. In a total of 169 NSCLC patients, only 40 (23.7%) patients exhibited positive CTC detection (>1 per 7.5 mL blood), in a similar agreement with that reported in western populations [15, 21–22].

**Table 1:** Characteristics of NSCLC patients.

Characteristics		
Gender	Male	112 (66.3%)
	Female	57 (33.7%)
Age	<60	71 (42.0%)
	≥ 60	98 (58.0%)
Histology	ADC	112 (66.3%)
	SCC	51 (30.2%)
	OC	6 (3.6%)
Smoking status	Never	69 (40.8%)
	Former	31 (18.3%)
	Current	69 (40.8%)
Location of primary tumor	Center	63 (37.3%)
	Peripheral	106 (62.7%)
Staging	I	14(8.3%)
	II	16 (9.5%)
	III	45 (26.6%)
	IV	94 (55.6%)
CTC counts	≥ 1	40 (23.7%)
	≥ 2	20 (11.8%)
	≥ 5	13 (7.7%)

ADC*: adenocarcinoma.

SCC*: squamous cell carcinoma.

OC*: other carcinomas, including large cell, mixed cell carcinoma, or undifferentiated carcinoma.

Intriguingly, our cohort samples exhibited a relatively low percentage of female patients (33.7%) as compared with western countries, where NSCLC is more prevalent in women [1–2]. It could be possible that differences in ethnicity and etiologic factors of NSCLC patients may account for this discrepancy. For example, the smoking rate is relatively low in Chinese females.

### Univariate analysis of CTC counts with clinical and pathological data

In correlation analyses, we found no significant association of CTC counts with gender, age, smoking status, and location of primary tumor (Table 2). However, histological type was positively associated with the CTCs (p<0.05 for CTC thresholds ≥ 1 and ≥ 2CTC/7.5 mL blood). No patients at stage I NSCLC had CTC ≥ 1 CTC/7.5 ml blood, while 15.8% (6/38) of stage II-IIIA patients and 29.1% (34/117) of stage IIIB-IV patient were found to have CTC ≥ 1 CTC/7.5 ml blood. Clinical TNM staging was positively associated with the CTCs, when it was divided into stage I, stage II, stage III and stage IV (p<0.05 for CTC thresholds ≥ 1 and 2 CTC/7.5 mL blood). Even when the Clinical TNM staging was divided into stage I-IIIA and IIIB-IV, it remained associated with CTCs (p<0.05 for CTC thresholds ≥1, 2 and 5 CTC/7.5 mL blood).

**Table 2:** Univariate analysis between CTC count (per 7.5 mL of peripheral blood) and clinico-pathological data.

Characteristic		<1 (%)	≥1 (%)	<2 (%)	≥2 (%)	<5 (%)	≥5 (%)
**Gender**	Male	85(75.9)	27(24.1)	99(88.4)	13(11.6)	104(92.9)	8(7.1)
	Female	44(77.2)	13(22.8)	50(87.7)	7(12.3)	52(91.2)	5(8.8)
*p*-value		1		1		0.763	
**Age**	<60	53(74.6)	18(25.4)	64(90.1)	7(9.9)	67(94.4)	4(5.6)
	≥60	76(77.6)	22(22.4)	85(86.7)	13(13.3)	89(90.8)	9(9.2)
*p*-value		0.715		0.631		0.561	
**Smoking**	Never	54(78.3)	15(21.7)	65(94.2)	4(5.8)	66(95.7)	3(4.3)
	Former	22(71.0)	9(29.0)	25(80.6)	6(19.4)	28(90.3)	3(9.7)
	Current	53(76.8)	16(23.2)	59(85.5)	10(14.5)	62(89.9)	7(10.1)
*p*-value		0.745		0.085		0.408	
**Histology**	ADC*	82(73.2)	30(26.8)	94(83.9)	18(16.1)	101(90.2)	11(9.8)
	SCC*	44(86.3)	7(13.7)	50(98.0)	1(2.0)	50(98.0)	1(2.0)
	OC*	3(50.0)	3(50.0)	5(83.3)	1(16.7)	5(83.3)	(16.7)1
*p*-value		0.049		0.014		0.118	
**Location**	Center	48(76.2)	15(23.80)	55(87.3)	8(12.7)	58(92.1)	5(7.9)
	Peripheral	81(76.4)	25(23.6)	94(88.7)	12(11.3)	98(92.5)	8(97.5)
*p*-value		1		0.809		1	
**Clinical staging**	I	14(100.0)	0(0.0)	14(100.0)	0(0.0)	14(100.0)	0(0.0)
	II	14(87.5)	2(12.5)	16(100.0)	0(0.0)	16(100.0)	0(0.0)
	III	36(80.0)	9(20.0)	43(95.6)	2(4.4)	43(95.6)	2(4.4)
	IV	65(69.1)	29(30.9)	76(80.9)	18(19.1)	83(88.3)	11(11.7)
*p*-value		0.03		0.013		0.253	
	I-IIIA	46(88.5)	6(11.5)	52(100.0)	0(0.0)	52(100.0)	0(0.0)
	IIIB-IV	83(70.9)	34(29.1)	97(82.9)	20(17.1)	104(88.9)	13(11.1)
*p*-value		0.018		0.001		0.01	
**T**	T1	23(71.9)	9(28.1)	27(84.4)	5(15.6)	31(96.9)	1(3.1)
	T2	58(87.9)	8(12.1)	62(93.9)	4(6.1)	63(95.5)	3(4.5)
	T3	12(70.6)	5(29.4)	15(88.2)	2(11.8)	16(94.1)	1(5.9)
	T4	28(65.1)	15(34.9)	36(83.7)	7(16.3)	37(86.0)	6(14.0)
*p*-value		0.025		0.255		0.256	
**N**	N0	28(84.8)	5(15.2)	30(90.9)	3(9.1)	31(93.9)	2(6.1)
	N1	18(81.8)	4(18.2)	21(95.5)	1(4.5)	21(95.5)	1(4.5)
	N2	42(73.7)	15(26.3)	52(91.2)	5(8.8)	56(98.2)	1(1.8)
	N3	37(69.8)	16(30.2)	42(79.2)	11(20.8)	44(83.0)	9(17.0)
*p*-value		0.392		0.171		0.392	
**M**	M0	64(85.3)	11(14.7)	73(97.3)	2(2.7)	73(97.3)	2(2.7)
	M1	65(69.1)	29(30.9)	76(80.9)	18(19.1)	83(88.3)	11(11.7)
*p*-value		0.018		0.001		0.04	

ADC*: adenocarcinoma.

SCC*: squamous cell carcinoma.

OC*: other carcinomas, including large cell, mixed cell carcinoma, or undifferentiated carcinoma.

TNM: Tumor Node Metastasis staging.

To further understand if tumor size, invasiveness, or lymphatic and distant metastasis have an impact on CTC count, the relationship between CTCs and TNM stage was also assessed. We found that 28.1% (9/32) of T1, 12.1% (8/66) of T2, 29.4% (5/17) of T3, and 34.9% (15/43) of T4 patients had CTCs ≥ 1 CTC/7.5 mL blood, constituting a significant correlation between tumor size and the presence of at least 1 CTC/7.5 mL blood. However, there was no correlation between other threshold CTC counts and the tumor size. Similarly, there was no relationship between any threshold CTC count and lymph nodes metastasis. However, distant metastasis was correlated with CTC counts at thresholds of ≥ 1, 2 and 5 CTCs/7.5 ml (p < 0.05).

### Multivariate analysis demonstrates that CTCs correlates with advanced stage disease

To enhance the prediction value of CTCs in NSCLC, we performed multivariate analysis include age, gender, smoking history, histology, clinical-staging and the location of the primary tumor. We found that patients with stage IIIB-IV disease had a high incidence of CTCs compared with those with stage I-IIIA. (P < 0.05; Table 3).

**Table 3:** Multivariate analysis of the CTC positive model with adjusted odds ratios and 95% CI.

Risk factors	Exp (B)	95% CI	P-value
**Gender**			
Female	1	Reference	-
Male	1.213	0.503–2.926	0.667
**Age**			
≥ 60	1	Reference	-
<60	1.051	0.483–2.286	0.899
**Smoking**			0.719
Never	1	Reference	-
Ever	1.492	0.515–4.324	0.461
Current	1.019	0.393–2.641	0.969
**Clinical-staging**			0.058
I-IIIA	1	Reference	-
IIIB- IV	2.745	1.036–7.270	0.042
**Histology**			
ADC*	1	Reference	-
SCC*	0.409	0.144–1.166	0.095
OC*	3.162	0.525–19.043	0.209
**Location of tumor**			
Center	1	Reference	-
Peripheral	0.727	0.309–1.710	0.466

ADC*: adenocarcinoma.

SCC*: squamous cell carcinoma.

OC*: other carcinomas, including large cell, mixed cell carcinoma, or undifferentiated carcinoma.

### Association of CTC counts with Ki-67 and tumor grade in NSCLC patients

Pathological analysis indicated that poorly differentiated tumors were correlated with higher CTC counts (CTCs ≥ 2 /7.5 mL blood, p < 0.05, Table 4), compared with the moderately differentiated tumors. Patients with Ki-67-positive tumors appeared to have higher CTC counts, but this did not reach statistical significance.

**Table 4:** Association between CTC count and Ki-67 and tumor grade data.

Characteristic		<1 (%)	≥1 (%)	<2 (%)	≥2(%)	<5(%)	≥5(%)
**Ki-67**	Negative	18(85.7)	3(14.3)	20(95.2)	1(4.8)	20(95.2)	1(4.8)
	Positive	31(75.6)	10(24.4)	35(85.4)	6(14.6)	36(87.8)	5(12.2)
	**P value**	0.541		0.406		0.654	
**Tumor grade**	Poorly differentiated	17(70.8)	7(29.2)	20(83.3)	4(16.7)	21(87.5)	3(12.5)
	Moderately differentiated	28(90.3)	3(9.7)	31(100.0)	0(0.0)	31(100.0)	0(0.0)
	**P value**	0.084		0.031		0.077	

### CTCs counts correlate with CEA

The tumor markers CEA, Cyfra21-1, CA19-9, CA-125, and SCC-Ag were also analyzed in NSCLC patients. Each serum marker was compared independently with CTC counts to identify potential relationships. The only marker found to be associated with CTCs was serum CEA. Elevated serum CEA was positively associated with CTC counts at thresholds ≥ 1, 2 and 5 CTCs/7.5 mL blood, as compared with normal CEA levels (Table 5).

**Table 5:** Association between CTC counts and tumor marker data from NSCLC patients.

Characteristic		< 1 (%)	≥ 1 (%)	< 2 (%)	≥ 2 (%)	<5 (%)	≥ 5 (%)
**CEA**	Elevated	40(64.5)	22(35.5)	46(74.2)	16(25.8)	51(82.3)	11(17.7)
	Normal	43(91.5)	4(8.5)	47(100.0)	0(0.0)	47(100.0)	0(0.0)
*p*-value		0.001		0		0.001	
**CA125**	Elevated	25(80.6)	6(19.4)	25(80.6)	6(19.4)	26(83.9)	5(16.1)
	Normal	32(82.1)	7(17.9)	35(89.7)	4(10.3)	37(94.9)	2(5.1)
*p*-value		1		0.32		0.228	
**CA199**	Elevated	11(73.3)	4(26.7)	11(73.3)	4(26.7)	12(80.0)	3(20.0)
	Normal	50(82.0)	11(18.0)	54(88.5)	7(11.5)	56(91.8)	5(8.2)
*p*-value		0.478		0.212		0.188	
**Cyfra21-1**	Elevated	38(69.1)	17(30.9)	43(78.2)	12(21.8)	47(85.5)	8(14.5)
	Normal	43(82.7)	9(17.3)	48(92.3)	4(7.7)	49(94.2)	3(5.8)
*p*-value		0.118		0.057		0.204	
**SCCA**	Elevated	4(66.7)	2(33.3)	5(83.3)	1(16.7)	5(83.3)	1(16.7)
	Normal	56(81.2)	13(18.8)	59(85.5)	10(14.5)	62(89.9)	7(10.1)
*p*-value		0.593		1		0.504	

Further statistical analysis was performed to investigate whether there was any correlation between the presence of CTCs, TNM staging, and serum concentration of CEA. After adjustment for TNM staging (I-IIIA and IIIB- IV), serum CEA was still positively associated with the presence of CTC (OR 95%CI = 4.263 [1.194–15.226], p = 0.026.

### Serum CEA increases the prediction power of CTCs in tumor aggressiveness

Detection of CTCs in our NSCLC patients was relatively low, with only 23.7% of patients showing >1 CTC per 7.5 mL blood. We thus proposed to examine if addition of a cheap clinical variable would enhance its power for disease prediction. For this, we used logistic regression analysis to analyze the ability of CTCs in combination with serum CEA in predicting NSCLC aggressiveness, including TNM staging (I-IIIA and IIIB- IV), Ki-67 level, and tumor grade. As seen in Table 6, we found that the prediction ability of late staging and Ki-67 were increased in the group with the combined CTC and CEA model as compared with the CTC model.

**Table 6:** Prediction of NSCLC aggressiveness and TNM staging with CTCs or/and CEA.

Characteristic		CTC	CTC/CEA
**Staging IIIB- IV**	Cox & Snell R^2^	0.062	0.265
	Nagelkerke R^2^	0.088	0.373
	Percentage Correct	68.8	75.2
*p*-value		0.008	0
**Ki67 positive**	Cox & Snell R^2^	0.011	0.167
	Nagelkerke R^2^	0.016	0.229
	Percentage Correct	63.3	75.5
*p*-value		0.452	0.011
**Poorly differentiated**	Cox & Snell R^2^	0.064	0.064
**tumor grade**	Nagelkerke R^2^	0.085	0.085
	Percentage Correct	62	62
*p*-value		0.069	0.191

## Discussion

Circulating tumor cells (CTCs) have been studied as potential biomarkers to improve NSCLC diagnosis, prognosis, treatment, and surveillance. However, to our knowledge, our study is the first to use the CellSearch system to evaluate CTCs in a large cohort of Chinese NSCLC patients.

In the univariate analysis, we found that CTC counts in NSCLC patients were associated with late stage, adenocarcinomas, poorly differentiated tumor grade, and elevated CEA levels. Using logistic regression analysis, we found that the combined CTCs and CEA model had a better prediction for the aggressiveness of NSCLC (late staging and Ki-67). Our data suggest that clinical variables, particularly serum CEA, can enhance the prediction power of CTCs in NSCLC patients.

Previous studies have reported that 30% of patients have >1 CTC per 7.5 ml of blood, as measured by CellSearch, and that 15% have five or more CTCs [15,21–22]. In our study, 23.7, 11.8 and 7.7% of patients demonstrated at least 1, 2 and 5 CTCs/7.5 ml blood, respectively. While these levels are somewhat lower than those in previously reported studies, different patient populations may account for these discrepancies. In particular, previous studies have included heterogeneous populations of metastatic and operable NSCLC.

Common prognostic factors in NSCLC include Ki-67 positivity and tumor grade. [25–28]. Tumor grade is correlated with the levels of apoptosis and cell proliferation in lung adenocarcinomas, with more rapid turnover of tumor cells in poorly differentiated tumors [29]. Consistent with this concept, we found that high CTC counts were associated with poorly differentiated tumors. In particular, a poorly differentiated tumor grade was significantly associated with CTC counts of ≥ 1 or 2 CTC/7.5 mL blood. Moreover, high Ki-67 staining in the primary tumor was also associated with high CTC counts at all thresholds, although these data were not statistically significant due to the small cohort size. The relationship between proliferation and CTCs in NSCLC will require further study using a larger cohort.

Previous studies have assessed the relationship between CTCs and tumor markers in solid tumors [30–32]. However, data from lung cancer patients are still lacking, particularly in Chinese population. We investigated the relationship between CTCs and commonly-used NSCLC tumor markers, including CEA, CA19-9, CA-125, SCC, and CYFRA21-1. Interestingly, our data show that serum CEA levels are correlated with CTC counts in NSCLC patients. CEA, a glycoprotein normally produced during fetal development, is present at very low level in the serum of healthy adults. Elevated serum levels of CEA are associated with the development, course, stage and prognosis of NSCLC [4–5, 12]. The data in our study suggest that the combination of CTC counts and serum CEA was associated with more aggressive NSCLC. However, it is not clear why other serum biomarkers do not have similar associations with CTCs in NSCLC patients. Further studies using larger sample sizes are necessary to determine whether it was useful to include these markers in the prediction model.

Despite the progress in recent year, CellSearch technology still has many limitations. Detection of CTCs by CellSearch system relies on cell surface markers such as EpCAM. Thus, it may potentially miss CTCs that do not express the target antigen [33–34]. Similarly, aggressive tumor cells lose epithelial markers due to epithelial-mesenchymal transition (EMT) and will not be detected by CellSearch. It should be noted that the field is moving away from CTC counts to molecular and functional characterization of CTCs and use of CTCs as liquid biopsies to test whether certain patients are more likely to benefit from center biological therapies [35–36]. For example, phenotypic characterization of CTCs by including a “tumor-specific” marker gene, like CEA by RT-PCR, has an advantage of enhancing sensitivity.

The CellSearch assay is very costly in China. The finding of our study may suggest a cost-effective method for NSCLC patients to decide who should run for this expensive assay. Tumor markers are always used as biomarkers for earlier diagnosis, treatment efficacy and prognosis in NSCLC [4–5, 12, 37–38]. As serum CEA is associated with CTCs, it may be possible to recommend the CellSearch assay for those NSCLC patients who are CEA-positive. Alternatively, we may explore whether the NSCLC patients with both positive CTC and elevated CEA are different from other subtypes in therapeutic response.

It should be emphasized that this study included only 169 NSCLC patients in the correlation analysis. In addition, due to the limit of serum samples, we could not complete all biomarker assays for every patient, leading to low statistical power for some variables in the stratified subgroups. We hope that continuous collection of tumor biopsies from this ongoing project will strength our findings.

In summary, a simple and reliable method for identifying NSCLC patients with CTCs would have great prognostic value. We found that the presence of CTCs is associated with late stage and poorly differentiated tumors, adenocarcinomas, and elevated CEA levels. The combination of CTC counts with serum CEA values was associated with more tumor aggressiveness in NSCLC patients. Long-term follow-up will be required to determine the further significance of CTCs in NSCLC.
